# Draft Whole Genome Sequence of *Bacillus pumilus* Strain 3-19, a Chemical Mutant Overproducing Extracellular Ribonuclease

**DOI:** 10.1128/genomeA.00724-14

**Published:** 2014-07-24

**Authors:** Vera Ulyanova, Raihan Shah Mahmud, Elena Dudkina, Valentina Vershinina, Olga Ilinskaya

**Affiliations:** Institute of Fundamental Medicine and Biology, Kazan (Volga region) Federal University, Kazan, Russia

## Abstract

Here, we present a draft genome sequence of *Bacillus pumilus* strain 3-19. It was derived from soil-isolated *B. pumilus* 7P using chemical mutagenesis and is characterized by elevated production of extracellular ribonuclease which is known to possess different biological activities with potential of applications in experimental research, medicine, and biotechnology.

## GENOME ANNOUNCEMENT

*Bacillus pumilus* strain 3-19 was derived from wild-type strain *Bacillus pumilus* 7P by screening on streptomycin-containing media followed by three-step selection of colonies which were resistant to increasing concentrations of antibiotic (10, 250, and 500 µg/ml) and were characterized by overproduction of extracellular ribonuclease. The obtained mutant possessed an almost 10-fold higher level of ribonuclease activity in comparison to the parent strain. Both features make the 3-19 strain suitable for industrial production of ribonuclease which can be used as a potential antitumor and antiviral agent (1, 2) and as a RNA-degrading tool in molecular biology. The 3-19 strain was patented in Russia in 2010 (3) and deposited in the Russian National Collection of Industrial Microorganisms (VKPM) under accession number B3833.

Whole-genome shotgun sequencing of *B. pumilus* 3-19 was performed on a 454 GS Junior system (Roche, Switzerland) with approximately 23-fold overall genome coverage. The sequencing 171,451 reads were assembled with GS De Novo Assembler (version May 2014) resulting in 39 contigs (>200 bp). A calculated genome size was 3,573,949 bp with a G+C content of 41.9 mol%. The genome was annotated using the NCBI Prokaryotic Genome Annotation Pipeline (PGAAP) version 2.6 (http://www.ncbi.nlm.nih.gov/genomes/static/Pipeline.html). A total of 3,563 genes and 3,363 coding sequences (CDS) were predicted and 77 RNAs were identified, including 72 tRNAs, 4 rRNAs, and 1 non-coding RNA (ncRNA). The total size of the assembly, G+C content, as well as the numbers of coding sequences and ribosomal, transport, and non-coding RNAs were in good agreement with respective figures for the parent strain of *B. pumilus* 7P (3,582,806 bp; 42 mol%; 3,460 CDSs; 77 RNAs, respectively), whose draft genome sequence was reported recently (4).

A brief comparison of two genomes made with RAST version 4.0 (5) revealed that 3,522 coding sequences (95% of total CDSs in the 3-19 strain) are identical in both strains while 179 CDSs (5%) have certain differences. Regulatory and coding regions of a gene encoding the extracellular ribonuclease have no modifications in the 3-19 strain compared with the 7P strain. A more-detailed comparative analysis of the parent and mutant genomes will provide further insight into the mechanism of acquired streptomycin resistance and the nature of ribonuclease overproduction.

### Nucleotide sequence accession number.

This whole-genome shotgun project has been deposited in DDBJ/EMBL/GenBank under the accession no. JOJX00000000. The version described in this paper is the first version.
